# A whole-genome transcriptome analysis of articular chondrocytes in secondary osteoarthritis of the hip

**DOI:** 10.1371/journal.pone.0199734

**Published:** 2018-06-26

**Authors:** Takashi Aki, Ko Hashimoto, Masanori Ogasawara, Eiji Itoi

**Affiliations:** Department of Orthopaedic Surgery, Tohoku University Graduate School of Medicine, Sendai, Japan; Ohio State University, UNITED STATES

## Abstract

**Objective:**

To date, exhaustive gene expression analyses of chondrocytes in hip osteoarthritis (OA) have yielded specific gene expression patterns. No study has reported on the exhaustive transcriptome of secondary hip OA based on acetabular dysplasia in a Japanese population, while previous reports have focused on primary or idiopathic hip OA in Caucasian populations. This study aims to search for specific gene expression patterns of secondary hip OA chondrocytes by transcriptome analysis.

**Design:**

Human articular cartilage was obtained from femoral heads following hemiarthroplasty for femoral neck fracture (N = 8; non-OA) and total hip arthroplasty for secondary hip OA (N = 12). Total RNA was extracted from the articular cartilage and submitted for microarray analysis. The obtained data were used to perform gene expression analysis, GO enrichment analysis and pathway analysis and were compared with data from primary hip OA in Caucasian populations in the literature.

**Results:**

We identified 888 upregulated (fold change: FC ≥ 2) and 732 downregulated (FC ≤ 0.5) genes in hip OA versus non-OA chondrocytes, respectively. Only 10% of upregulated genes were common between the secondary and primary OA. The newly found genes prominently overexpressed in the secondary hip OA chondrocytes were *DPT*, *IGFBP7*, and *KLF2*. Pathway analysis revealed extracellular matrix (ECM)-receptor interaction as an OA-related pathway, which was similar to previous reports in primary hip OA.

**Conclusions:**

This is the first study to report the genome-wide transcriptome of secondary hip OA chondrocytes and demonstrates new potential OA-related genes. Gene expression patterns were different between secondary and primary hip OA, although the results of pathway and functional analysis were similar.

## Introduction

Osteoarthritis (OA) is a joint disease characterized by irreversible, degenerative changes in joint components such as articular cartilage, synovium, and subchondral bone [1]. It is one of the leading causes of disability occurring mainly in the elderly due to severe joint pain and deformity especially in weight-bearing joints such as the knee and hip. Approximately 10–15% of the world’s population suffers from symptomatic OA [2]. In Japan, 25 and 12 million people were estimated to suffer from knee OA and hip OA, respectively [3, 4]. The prevalence of OA increases yearly as the number of elderly rise. Although OA is a global “common disease” as mentioned above, no definitive therapy has been developed to alleviate degraded joint components. Once joint destruction is at the end stage, surgeries such as arthroplasty or joint replacement are the only solution to relieve joint pain and deformity. These days, medical services for OA are giving a huge socioeconomical impact on increasing medical costs [5].

OA develops due to various causes including aging, obesity, mechanical load and genetic factors. Previous studies have investigated a genetic influence in OA [6] and suggested that genetic susceptibility of OA varies by joints, such as the hip or knee [7]. Furthermore, hip OA is more influenced by genetic factors than are other joints. Recently, genetic research has developed rapidly with novel sequencing technologies and microarray analysis [8]. Exhaustive transcriptome analysis of chondrocytes in primary hip OA has uncovered some specific gene expression patterns. Idiopathic OA, also known as primary OA is the dominant form of hip OA in Caucasians [9]. Meanwhile in Japan, approximately 80–90% of hip OA is considered to be secondary following acetabular dysplasia (AD) [10, 11]. Considering the mechanisms of onset and progression, primary and secondary hip OA should have different pathomechanisms. In this respect, gene expression patterns in the chondrocytes of primary hip OA in previous reports are assumed to be different from those in secondary OA Japanese patients. Most exhaustive gene analyses have been targeted to primary OA [12, 13]; however, no exhaustive gene analysis has been conducted yet in patients with secondary hip OA. Therefore, this study aims to identify specific gene expression patterns with microarray analysis of the articular chondrocytes on secondary hip OA in Japanese population. Furthermore, differences or similarities in the transcriptome of OA chondrocytes between the primary and secondary are discussed and compared with previous reports.

## Materials and method

### Ethics statement

This study was approved by the Ethical Review Board of Tohoku University Graduate School of Medicine. Informed consent was obtained from all patients for the use of study data.

### Human articular cartilage harvesting

Human articular cartilage pieces were obtained after hemiarthroplasty following fracture of the neck of femur (#NOF: non-OA), or total hip arthroplasty for secondary hip OA. Surgeries were performed at affiliated hospitals of the Department of Orthopaedic Surgery, Tohoku University, Sendai, Japan. Clinical findings for each patient were recorded by survey form including medical history, medication history, and radiographic classification of OA [14]. Radiological evaluation of hip OA was performed using the Japanese Orthopaedic Association staging criteria. The center-edge angle, the angle formed by a vertical line and a line drawn from the center of the femoral head to the edge of the acetabulum on plain X-ray, <20 degrees was defined as AD of the hip [10]. All OA patients in this study were in the terminal stage of secondary hip OA based on AD. Patients with primary hip OA, rheumatoid arthritis, or osteonecrosis of the hip were excluded from the study.

### RNA extraction

**Fig 1:**
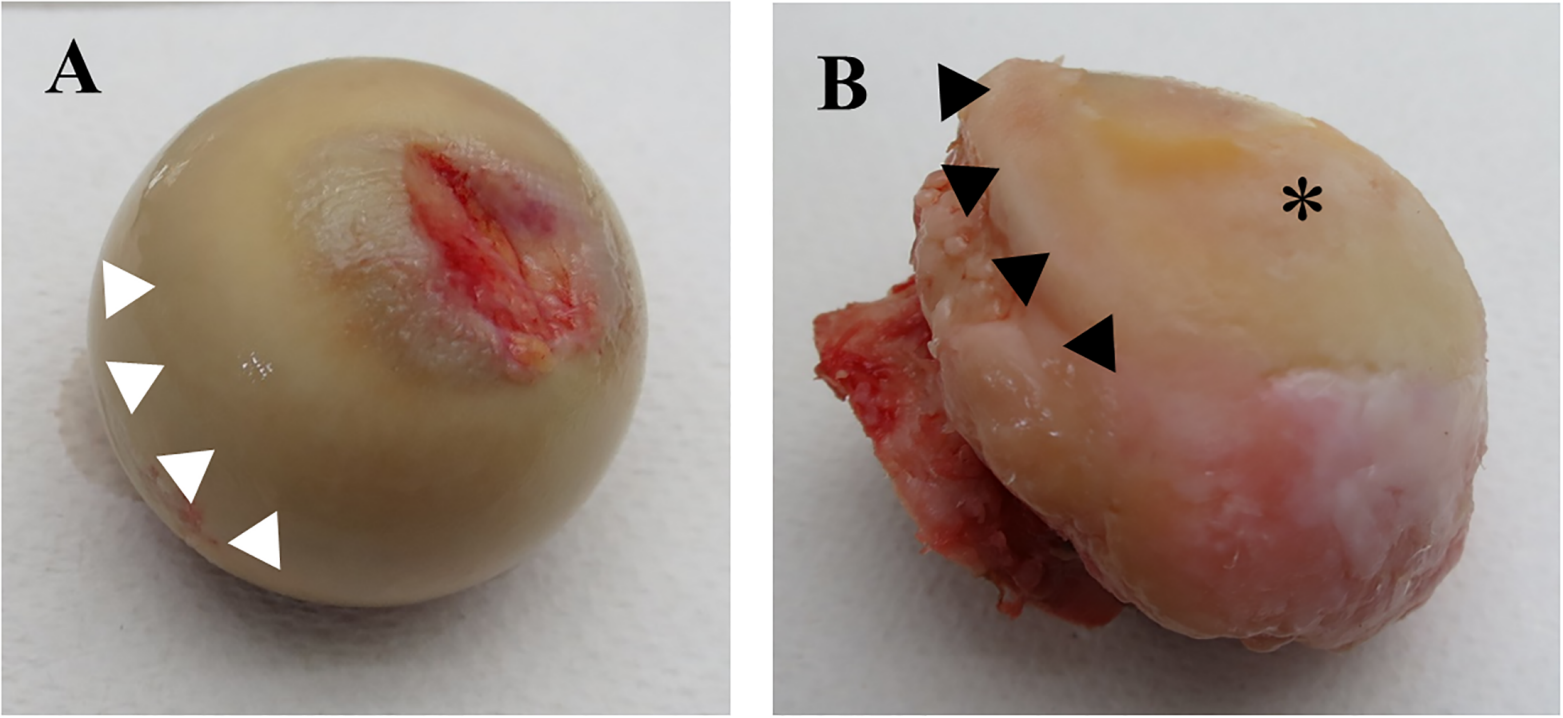
Femoral head of #NOF (fracture of neck of femur: non-OA) and OA. (A) A femoral head of #NOF. (B) A femoral head of a typical secondary OA. The articular cartilage pieces were obtained from the middle to deep layer of the non-weight bearing, macroscopically intact area from #NOF femoral head (white arrow heads), and the surface layer (black arrow heads) surrounding the weight-bearing, eburnated area (*) from OA femoral heads, respectively.

Articular cartilage pieces were harvested from femoral heads within 6 hours of surgery. Non-OA cartilage samples were obtained from the middle to deep zone of microscopically intact, non weight-bearing part of #NOF femoral heads as shown in the previous study [15]. OA cartilage samples were harvested from severely eroded areas of femoral head cartilage next to the weight-bearing area (Fig 1).

Cartilage pieces were finely diced and suspended in QIAzol^®^ Lysis Reagent (Qiagen, Crawley, UK). The suspension was homogenized on ice with TissueRuptor^™^ (Qiagen, Crawley, UK) for efficient disruption of the cartilage pieces. Total RNA was directly extracted from the supernatant prepared above using the Qiagen RNeasy Lipid Tissue Mini Kit^®^ (Qiagen, Crawley, UK) according to the manufacturer’s instructions. Concentration and quality of total RNA were assessed by NanoDrop^™^ Lite (Thermo Fisher Scientific, Inc., Waltham, MA, USA) and Agilent 2100 Bioanalyzer^®^ (Agilent Technologies, Santa Clara, CA, USA), respectively. Extracted total RNA was also reverse transcribed with High Capacity cDNA Reverse Transcription Kit (Applied Biosystems, CA, USA) immediately following extraction for quantitative real-time polymerase chain reaction (qRT-PCR).

### Transcriptome analysis with microarray

Extracted total RNA from 8 non-OA and 12 OA chondrocytes with RNA Integrity Number ≥ 6.5 underwent microarray analysis using 3D-Gene Human Oligo Chip 25k (Toray Industries Inc., Tokyo, Japan). Demographic data of the samples and quality of RNA are shown in Table 1. Extracted total RNA was labeled with Cy5 using the Amino Allyl MessageAmp^™^ II aRNA Amplification Kit (Applied Biosystems, CA, USA). The Cy5-labeled aRNA pools were applied to a hybridization buffer and hybridized for 16 h following the supplier’s protocols (www.3d-gene.com). Fluorescent signals were scanned using a 3D-Gene Scanner (Toray Industries Inc., Tokyo, Japan) and analyzed with 3D-Gene Extraction software (Toray Industries Inc., Tokyo, Japan). A global normalization method was used to adjust the median of all detected signal intensities to 25. The genes were selected for further analysis when (1) expression with a mean fold change (FC) of ≤ 0.5 or ≥ 2 between the non-OA and OA groups was observed and (2) the round-robin comparison of the gene expression between the 8 non-OA and 12 OA samples (96 combinations in total) showed a significant difference in > 80% (≥ 77 of 96) of the combinations. The analysis aims to narrow down the genes with higher specificity without affecting the average value of gene expression according to the previous report [16]. A *p*-value < 0.01 was considered statistically significant.

**Table 1:** The demographic data of the cartilage samples of non-OA and OA.

**Microarray**		
	Non-OA (n = 8)	OA (n = 12)
Mean age ± SD (years)	80 ± 7	64 ± 7
Male: Female	1: 7	1: 11
Quality of RNA		
Mean RIN ± SD	7.3 ± 0.8	7.4 ± 0.5
Mean A260/280 ± SD	1.9 ± 0.04	1.9 ± 0.1
**qRT-PCR**		
	Non-OA (n = 6)	OA (n = 6)
Mean age ± SD (years)	85 ± 7	71 ± 11
Male: Female	1: 5	1: 5

OA, osteoarthritis; RIN, RNA integrity number; qRT-PCR, Quantitative reverse transcriptase polymerase chain reaction; SD, Standard deviation.

Gene Ontology (GO) functional enrichment analysis for the differentially expressed genes was performed by Gene Set Enrichment Analysis software (http://software.broadinstitute.org/gsea/index.jsp) [17]. The gene sets were separated according to the GO terms for biological processes, cellular components, and molecular functions. A *p*-value < 0.01 and false discovery rate (FDR) *q*-values ≤ 0.25 were used to filter results. Pathway analysis was performed using GeneCodis tools (http://genecodis.cnb.csic.es/) based on the Kyoto Encyclopedia of Genes and Genomes (KEGG) pathway database with a *p*-value < 0.05 considered statistically significant [18].

### Quantitative real-time polymerase chain reaction (qRT-PCR)

To replicate the gene expression profiles in the microarray analysis, total RNA extracted from 6 independent non-OA and 6 OA samples were analyzed by qRT-PCR. Twelve differently expressed genes in OA chondrocytes, *ASPN*, *COL1A2*, *COL2A1*, *COL3A1*, *COL5A2*, *KLF2*, *MXRA5*, *OGN*, *SPARC*, *TGFBI*, and *TNFAIP6*, were selected according to our microarray data with the following conditions: (1) mean FC ≥ 10 in OA compared to non-OA groups, (2) expression signals ≥ 1000 in OA, and (3) > 80% (≥ 77 of 96 combinations) of the round-robin comparisons between 8 non-OA and 12 OA samples showed significantly increased expression in OA. Relative quantification of gene expression was performed with an Applied Biosystems^®^ StepOnePlus^™^ Real-time PCR System (Applied Biosystems, CA, USA) using TaqMan^®^ Gene Expression Assays (Applied Biosystems). Reactions were performed in duplicate with glyceraldehyde-3-phosphate dehydrogenase (*GAPDH*) as an internal control gene. A 20-μL reaction mixture was prepared for each reaction containing 1 μL of complementary DNA, 10 μL of TaqMan^®^ Universal PCR Master Mix (Applied Biosystems), 1 μL of premixed gene assay mix with 5 μM of TaqMan^®^ probe and 18 μM for each primer, and 8 μL of nuclease-free water. Thermocycler conditions consisted of an initial activation at 95°C for 10 minutes, followed by a 2-step PCR program at 95°C for 15 seconds and 60°C for 60 seconds for 40 cycles. The 2^-ΔΔCt^ method was used for relative quantification of gene expression. A dissociation curve was obtained after each qPCR run to validate reactions. Data are shown in average ± standard error of the mean. Statistical analysis for qPCR data was performed using the double-sided Mann–Whitney U test with IBM^®^ SPSS^®^ Statistics version 21.0 software. A *p*-value < 0.05 was considered statistically significant.

### Histological analysis

Full-thickness human articular cartilage specimens were harvested from the femoral heads of the non-OA and OA group, respectively. The cartilage was fixed with 4% paraformaldehyde solution in PBS and embedded in paraffin. Samples were cut into 7-μm sections and deparaffinized in xylene and dehydrated in gradually diluted ethanol from 100% to 70%. safranin-O and alcian blue/sirius red staining were performed to evaluate cartilage morphology [19].

### Comparative analysis between Japanese secondary OA and Caucasian primary OA microarrays

Microarray data of Japanese hip OA were compared with the data by Xu et al [13] obtained from North European populations of UK citizens. In the study, total RNA was extracted from femoral heads of 10 #NOF (non-OA) and 9 hip OA patients in a similar manner. Gene expression profiling was assessed by Illumina Whole-genome Expression Array Human HT-12 V3 (Illumina Inc., Saffron Walden, UK). Differentially expressed genes between non-OA and OA (FC ≤ 0.5 and ≥ 2, *p* < 0.01) were extracted from Xu’s study to match microarray data for comparison. The upregulated and downregulated genes were compared with our data. Furthermore, a pathway analysis using KEGG on the differentially expressed genes was conducted to assess potentially active pathways in OA chondrocytes between the Caucasian and Japanese populations.

## Results

### Gene expression profiles of non-OA and OA

Microarray analysis identified 888 upregulated (FC ≥ 2) and 732 downregulated (FC ≤ 0.5) genes in OA chondrocytes compared to non-OA chondrocytes (*p* < 0.01), respectively. Round-robin comparison of differentially expressed gene expression between 8 non-OA and 12 OA samples (96 combinations in total) revealed 352 upregulated and 159 downregulated genes in OA chondrocytes in > 80% of 96 combinations (S1 Table). Among these, expression levels of 65 genes and 12 genes were > 10 times more (FC ≥ 10) and less (FC ≤ 0.1) in OA compared to non-OA chondrocytes, respectively. The most differentially expressed genes between non-OA and OA are shown in Table 2. *COL1A2* gene showed the most prominent upregulation in OA chondrocytes with FC > 700. The most downregulated genes in OA were *APOD* (apolipoprotein D) and *DIO3* (iodothyronine deiodinase type III) showing FC < 0.02. In total, 18 transcription factor (TF) genes, (*ARNTL2*, *DLX3*, *EGR2*, *FOXO1*, *GLI3*, *HEY2*, *JDP2*, *KLF2*, *MSX1*, *NR4A1*, *SIX3*, *TFDP1*, *TRPS1*, *ZNF304*, *ZNF544*, *ZNF746*) were upregulated and 8 (*BCL6*, *ELF3*, *FLI1*, *HIF3A*, *NR1I3*, *NR3C2*, *PAX8*, *ZNF302*) were downregulated in OA. Among them, KLF2 (Krüppel-like factor 2) was the most prominently overexpressed in OA.

**Table 2:** Representative differentially expressed genes in OA and NOF cartilage.

Gene symbol	Gene name	Public ID	Fold-change (OA vs NOF)	*P*-value
*Up-regulated*				
**COL1A2**	**collagen type I alpha 2**	NM_000089.3	759.21	0.00021
**ASPN**	**asporin**	NM_001193335.1	177.05	1.98E-06
**TPPP3**	**tubulin polymerization-promoting protein family member 3**	NM_016140.3	122.19	1.71E-06
OGN	osteoglycin	NM_024416.4	110.06	2.41E-06
**COL3A1**	**collagen type III alpha 1**	NM_000090.3	80.88	9.22E-07
MXRA5	matrix-remodelling associated 5	NM_015419.3	58.80	1.97E-08
**DPT**	**dermatopontin**	NM_001937.4	58.23	2.44E-06
**AQP1**	**aquaporin 1 (Colton blood group)**	NM_001185061.1	50.87	0.00048
CRIP1	cysteine rich protein 1	NM_001311.4	47.59	1.38E-06
**TGFBI**	**transforming growth factor beta induced**	NM_000358.2	37.35	7.38E-06
**MINOS1-NBL1**	**MINOS1-NBL1 readthrough**	NM_001204089.1	36.83	4.90E-05
**S100A4**	**S100 calcium binding protein A4**	NM_019554.2	35.65	1.86E-08
COL2A1	collagen type II alpha 1	XM_011537935.1	31.45	1.43E-06
**TMSB4X**	**thymosin beta 4, X-linked**	NM_021109.3	23.63	0.00014
**CRLF1**	**cytokine receptor-like factor 1**	NM_004750.4	23.62	6.27E-06
COL5A2	collagen type V alpha 2	NM_000393.3	18.72	1.03E-08
**IGFBP7**	**insulin like growth factor binding protein 7**	NM_001553.2	16.60	1.99E-07
**ANOS1**	**anosmin 1**	NM_000216.2	16.19	7.47E-05
PCOLCE	procollagen C-endopeptidase enhancer	NM_002593.3	14.53	2.28E-07
SPARC	secreted protein acidic and cysteine rich	NM_001309443.1	11.51	1.05E-11
**DIO2**	**deiodinase, iodothyronine, type II**	NM_000793.5	11.17	0.00047
**KLF2**	**Kruppel-like factor 2**	NM_016270.2	11.12	0.00011
**TNFAIP6**	**TNF alpha induced protein 6**	NM_007115.3	10.90	0.00014
*Down-regulated*				
**APOD**	**apolipoprotein D**	NM_001647.3	0.018	5.07E-06
**DIO3**	**deiodinase, iodothyronine, type III**	NM_001362.3	0.020	0.0046
LCN2	lipocalin 2	NM_005564.4	0.026	2.50E-06
**C4BPA**	**complement component 4 binding protein alpha**	NM_000715.3	0.043	0.00027
C10orf10	chromosome 10 open reading frame 10	NM_007021.3	0.053	0.000024
CCL20	C-C motif chemokine ligand 20	NM_001130046.1	0.063	0.0093
**CP**	**ceruloplasmin (ferroxidase)**	NR_046371.1	0.066	0.000024
STEAP4	STEAP4 metalloreductase	NM_024636.3	0.072	0.00073
HIST1H1C	histone cluster 1, H1c	NM_005319.3	0.072	0.00022
BEX2	brain expressed X-linked 2	NM_001168401.1	0.074	2.80E-07
**GPX3**	**glutathione peroxidase 3**	NM_002084.3	0.086	0.000014
**PDZK1IP1**	**PDZK1 interacting protein 1**	NM_005764.3	0.088	0.0026

Representative differentially expressed genes in OA and #NOF cartilage (Fold change ≤ 0.1 or ≥ 10, ≥ 80% significantly expressed by round-robin analysis and gene expression signal ≥1000 for up-regulated gene). Uniquely expressed gene in our secondary OA chondrocytes compared to primary one [13] are shown in bold letters.

### Functional enrichment analysis

The dominant function of differentially expressed genes within the 3 GO categories (cellular component, biological process, and molecular function) was evaluated. The differentially expressed genes identified 23 upregulated and 3 downregulated GO terms, respectively (Table 3). The core enrichment genes in each significant function are listed in S2 Table. Biological process, cellular components, and molecular functions identified 6, 10, and 6 upregulated functions and 1, 0, and 2 downregulated functions, respectively. Within all categories, extracellular matrix (ECM) belonging to “cellular components” contained 61 differentially upregulated genes and was significant (FDR-*q* = 0.069).

**Table 3:** Functional enrichment analysis using a gene set enrichment analysis (GSEA).

Gene Ontology name	Number of genes	*P*-value	FDR
**Biological process_UP**			
Sensory organ development	23	<0.001	0.123
Collagen fibril organization	10	<0.001	0.077
Response to growth factor	20	<0.001	0.201
Ear development	11	<0.001	0.164
Extracellular structure organization	42	<0.001	0.17
Heart development	23	<0.001	0.162
**Biological process_DOWN**			
Gamete generation	13	<0.001	0.216
**Cellular components_UP**			
Extracellular matrix	61	<0.001	0.069
Proteinaceous extracellular matrix	52	0.014	0.211
Endoplasmic reticulum lumen	19	0.013	0.192
Cell cortex	10	0.016	0.237
Basement membrane	17	0.045	0.209
Cell leading edge	14	<0.001	0.203
Cell substrate junction	15	0.048	0.213
Collagen trimer	14	0.018	0.197
Anchoring junction	15	0.014	0.208
Endoplasmic reticulum	55	0.042	0.211
**Cellular components_DOWN**			
None			
**Molecular functions_UP**			
Growth factor binding	14	0.015	0.21
Tracellulra matrix structural constituent	15	0.029	0.217
Prptein complex binding	31	<0.001	0.212
Cell adhesion molecule binding	11	0.031	0.238
Transcription factor binding	11	0.03	0.21
Identical protein binding	34	0.041	0.204
**Molecular functions_DOWN**			
Cation transmembrane transporter activity	13	0.045	0.183
Transporter activity	29	<0.001	0.225

Dominant functions (*p* < 0.05 and FDR-*q* < 0.25) are listed in the table.

### Pathway analysis

Pathway analysis revealed 51 upregulated pathways in Japanese hip OA (*p* < 0.05) (Table 4 and S3 Table). Overall, 47% of the genes composing each of the ECM-receptor interaction, focal adhesion, and protein digestion and absorption pathways were the differentially expressed in OA chondrocytes.

**Table 4:** The upregulated pathways in OA chondrocytes.

pathway	*P*-value	Ratio
(KEGG) 00052: Galactose metabolism	0.0019	0.11
(KEGG) 00480: Glutathione metabolism	0.0075	0.07
(KEGG) 03320: PPAR signaling pathway	0.026	0.04
(KEGG) 04010: MAPK signaling pathway	0.038	0.04
(KEGG) 04060: Cytokine-cytokine receptor interaction	0.00088	0.14
(KEGG) 04080: Neuroactive ligand-receptor interaction	0.00084	0.03
(KEGG) 04110: Cell cycle	0.00025	0.21
(KEGG) 04115: p53 signaling pathway	0.0013	0.13
(KEGG) 04144: Endocytosis	0.0087	0.03
(KEGG) 04145: Phagosome	8.52E-05	0.30
(KEGG) 04310: Wnt signaling pathway	0.0029	0.06
(KEGG) 04340: Hedgehog signaling pathway	0.0030	0.09
(KEGG) 04350: TGF-beta signaling pathway	0.00088	0.14
(KEGG) 04360: Axon guidance	0.0064	0.04
(KEGG) 04510: Focal adhesion	9.66E-13	0.47
(KEGG) 04512: ECM-receptor interaction	9.66E-13	0.47
(KEGG) 04514: Cell adhesion molecules (CAMs)	8.52E-05	0.30
(KEGG) 04540: Gap junction	0.00046	0.18
(KEGG) 04612: Antigen processing and presentation	8.52E-05	0.30
(KEGG) 04640: Hematopoietic cell lineage	0.0070	0.05
(KEGG) 04670: Leukocyte transendothelial migration	0.020	0.04
(KEGG) 04672: Intestinal immune network for IgA production	8.52E-05	0.30
(KEGG) 04810: Regulation of actin cytoskeleton	0.043	0.02
(KEGG) 04940: Type I diabetes mellitus	8.52E-05	0.30
(KEGG) 04974: Protein digestion and absorption	9.66E-13	0.47
(KEGG) 04976: Bile secretion	0.027	0.04
(KEGG) 05130: Pathogenic Escherichia coli infection	0.00046	0.18
(KEGG) 05140: Leishmaniasis	8.52E-05	0.30
(KEGG) 05145: Toxoplasmosis	8.52E-05	0.30
(KEGG) 05146: Amoebiasis	7.29E-08	0.38
(KEGG) 05150: Staphylococcus aureus infection	8.52E-05	0.30
(KEGG) 05152: Tuberculosis	8.52E-05	0.30
(KEGG) 05200: Pathways in cancer	0.00025	0.21
(KEGG) 05210: Colorectal cancer	0.0046	0.08
(KEGG) 05212: Pancreatic cancer	0.00025	0.21
(KEGG) 05215: Prostate cancer	0.046	0.03
(KEGG) 05217: Basal cell carcinoma	0.0030	0.09
(KEGG) 05218: Melanoma	0.0038	0.06
(KEGG) 05219: Bladder cancer	0.00057	0.10
(KEGG) 05220: Chronic myeloid leukemia	0.00025	0.21
(KEGG) 05222: Small cell lung cancer	0.0066	0.07
(KEGG) 05310: Asthma	8.52E-05	0.30
(KEGG) 05320: Autoimmune thyroid disease	8.52E-05	0.30
(KEGG) 05322: Systemic lupus erythematosus	8.52E-05	0.30
(KEGG) 05323: Rheumatoid arthritis	8.52E-05	0.30
(KEGG) 05330: Allograft rejection	8.52E-05	0.30
(KEGG) 05332: Graft-versus-host disease	8.52E-05	0.30
(KEGG) 05410: Hypertrophic cardiomyopathy (HCM)	0.0045	0.05
(KEGG) 05412: Arrhythmogenic right ventricular cardiomyopathy (ARVC)	0.029	0.04
(KEGG) 05414: Dilated cardiomyopathy	0.0045	0.05
(KEGG) 05416: Viral myocarditis	8.52E-05	0.30

The ratio is calculated by dividing the number of overexpressed genes in OA chondrocytes by the total number of genes contained in the pathway. Pathways overlapped in the comparative analysis between our secondary OA data and primary one [13] are shown in bold letters.

### qRT-PCR

**Fig 2:**
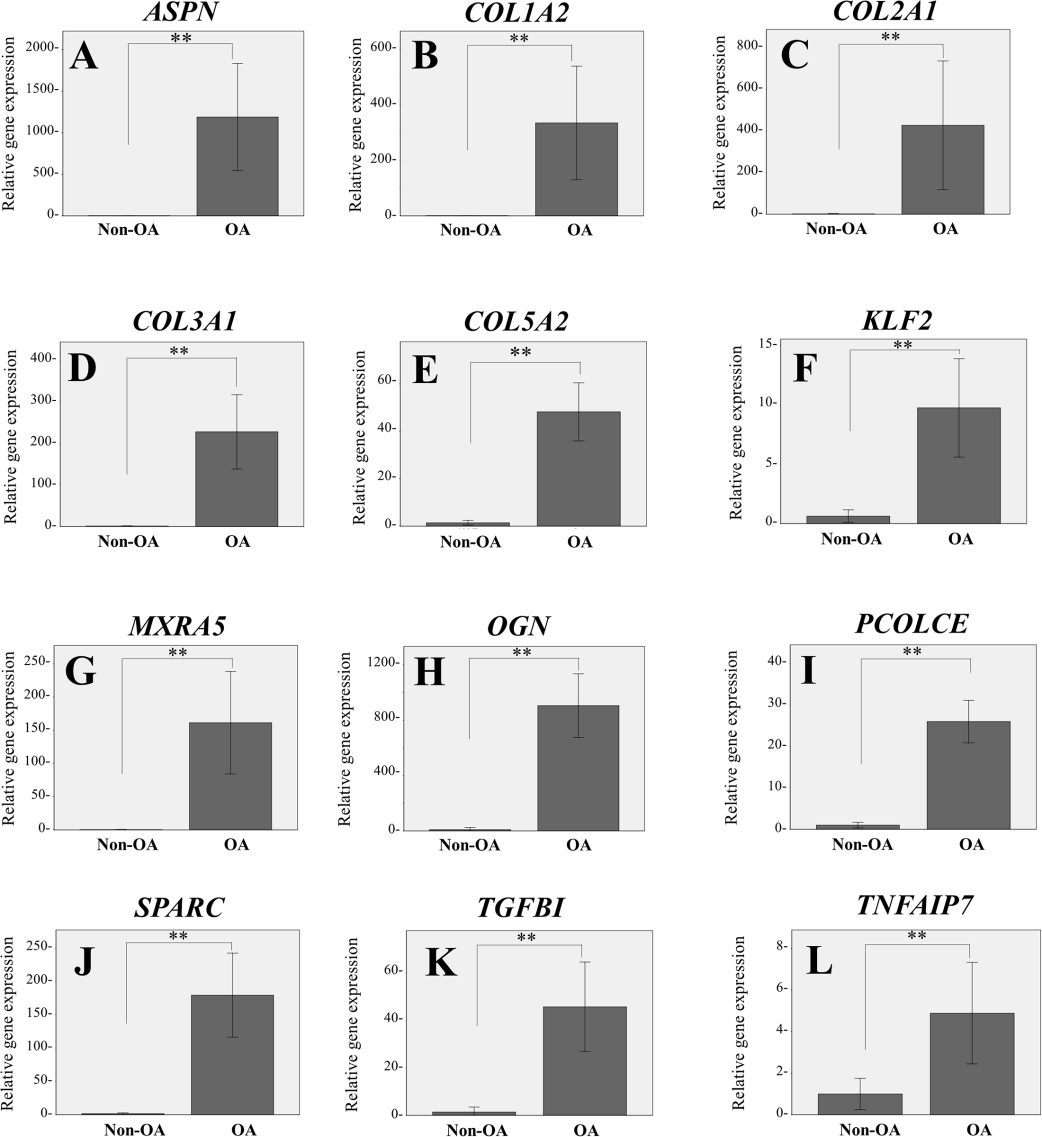
qRT-PCR replication study. The figures show relative gene expression of 12 prominently overexpressed gene in OA chondrocytes detected by the microarray analysis. *ASPN* (A), *COL1A2* (B), *COL2A1* (C), *COL3A2* (D), *COL5A1* (E), *KLF2* (F), *MXRA5* (G), *OGN* (H), *PCOLCE* (I), *SPARC* (J), *TGFBI* (K), and *TNFAIP6* (L). The data were shown as average ± standard error of mean (SEM) (**: *p* < 0.01).

To replicate the expression profiles obtained by microarray analysis, qRT-PCR performed relative quantification of gene expression in individual samples. The differentially expressed genes in OA chondrocytes in microarray analysis, *ASPN*, *COL1A2*, *COL2A1*, *COL3A1*, *COL5A2*, *KLF2*, XRA5, *OGN*, *PCOLCE*, *SPARC*, *TGFBI*, and *TNFAIP6*, demonstrated expression patterns similar to those found on microarray analysis (Fig 2A–2L).

### Histological analysis

**Fig 3:**
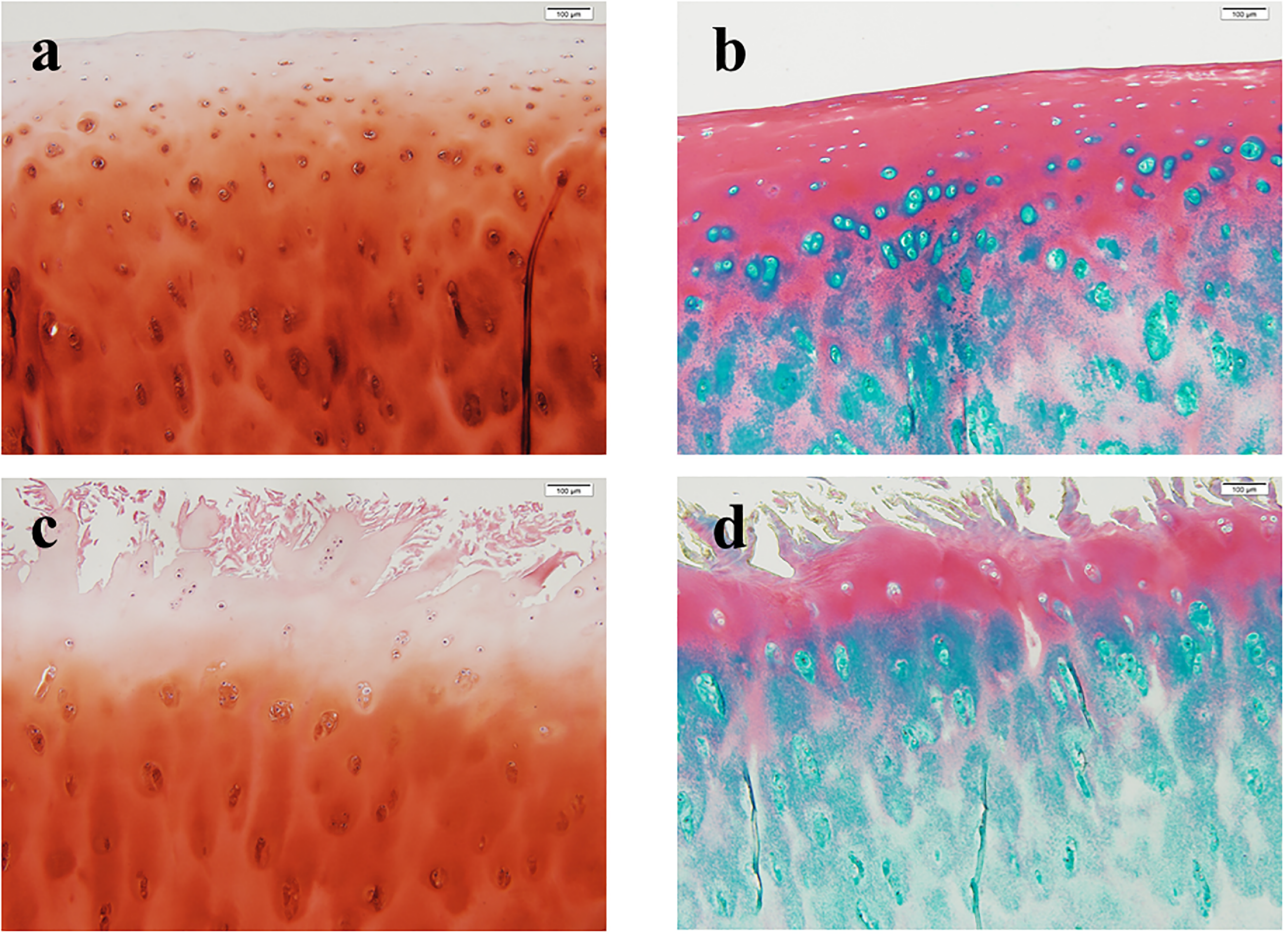
Historical image of articular cartilage. The figure shows the representative sections of non-OA (a, b) and OA (c, d) articular cartilage. Safranin O (a, c) and Alcian Blue / Sirius Red staining (b, d) were performed. On the surface layer of OA cartilage, degeneration with fibrillation and cracks was confirmed. Chondrocytes were enlarged and clusters formed. All figure taken with a magnification x100 and Scale bars = 100μm.

The histological images of representative samples of each group are shown in Fig 3. In non-OA cartilage, a thick ECM with a smooth surface surrounds sparsely located, flat-shaped chondrocytes. In OA cartilage, round-shaped chondrocytes form clusters in eroded ECM with an irregular surface. Depletion of glycosaminoglycans and collagen fibers in the ECM of OA cartilage was demonstrated by safranin-O and alcian blue/sirius red staining, respectively.

### Comparative study between secondary and primary hip OA

**Fig 4:**
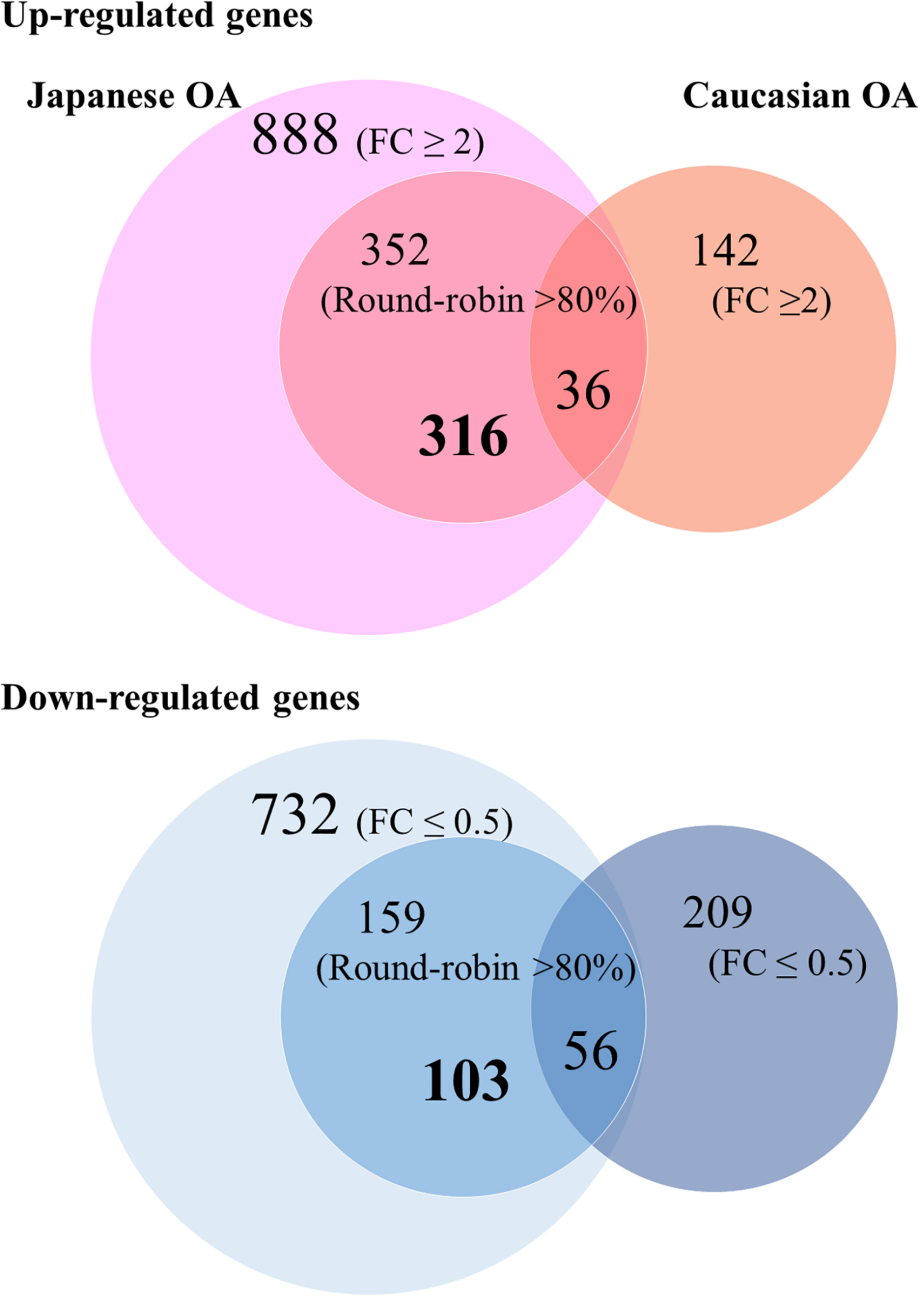
Compared gene profiles. Venn diagram demonstrating the overlap of the differentially expressed genes between secondary and primary hip OA. Overlapping portion of the three circles indicates the genes significantly expressed in both studies in common (36 up-regulated genes and 56 down-regulated genes). In the inner circles on the left side, the area not overlapped with the primary OA’s circle indicate differentially expressed genes in secondary (316 up-regulated genes and 103 down-regulated genes).

Microarray data of Xu et al [13] identified 142 upregulated (FC ≥ 2) and 209 downregulated (FC ≤ 0.5) genes, respectively (*p* < 0.01) in primary hip OA. Only 36 (10% of 352) upregulated genes and 56 (35% of 147) downregulated genes were overlapped between our secondary OA and their primary OA data (Fig 4). Among them, markedly expressed genes were *ASPN*, *COL1A2*, *COL3A1*, *DPT* (dermatopontin), *S100A4* (S100 calcium binding protein A4), and *TGFB1* (transforming growth factor beta). KEGG pathway analysis revealed that 6 out of 7 upregulated pathways, including focal adhesion, ECM-receptor interaction, and protein digestion and absorption pathway, were commonly upregulated between secondary and primary hip OA patients.

## Discussion

To the best of our knowledge, our study is the first whole-genome transcriptome analysis of articular chondrocytes in Japanese individuals with secondary hip OA. We also compared these data with transcriptome in a different ethnic origin. Few studies have reported on genome-wide transcriptome analysis of OA chondrocytes to date, presumably because extraction of high-quality nucleic acids from chondrocytes in articular cartilage is relatively difficult due to the amount of ECMs surrounding sparsely located chondrocytes [20]. Therefore, it is valuable to observe phenotypes of chondrocytes as they are in the articular cartilage.

Gene expression profiling of chondrocytes in secondary hip OA yielded 888 upregulated and 732 downregulated genes in OA chondrocytes compared to non-OA in contrast to primary OA data [13] demonstrating 142 upregulated and 209 downregulated genes. In our microarray analysis, highly upregulated genes in OA chondrocytes included a group of genes significant for ECM function by GO term, i.e., *ASPN*, *COL1A2*, *COL2A1*, *COL3A1*, *COL5A2*, *DPT*, *IGFBP7*, *OGN*, *PCOLCE*, and *SPARC* (Table 2). Compared to the primary OA data [13], only 10% of upregulated genes were overlapped (indicated in bold in Table 2 and S1 Table). Most commonly upregulated genes in OA chondrocytes were ECM-related genes including *COL2A1*, *COL5A2*, *OGN*, *PCOLCE*, and *SPARC*.

We found that *ASPN* was one of the most overexpressed genes in OA cartilage (FC = 17.05, *p* = 1.98E-06). Asporin, the protein encoded by *ASPN*, is an ECM component of the small leucine-rich proteoglycan family [21]. Asporin binds to collagen fiber to induce mineralization and inhibits TGF-β in cartilage differentiation [22]. Considering the facts above, *ASPN* can be potentially related to OA, particularly in patients with secondary OA. Indeed, a polymorphism in the *ASPN* gene is related to OA pathogenesis in the Japanese population [23]. Moreover, the genome copy number variation of *ASPN* can cause severe AD, a major cause of hip OA in Japan [24]. Our results support those previous studies conducted in Asian populations. Conversely, some studies stated that *ASPN* does not influence OA etiology in UK Caucasians [25]. These facts indicate racial differences in genetic susceptibility of OA. As most secondary hip OA in Asians population is caused by AD [11], *ASPN* could be an important factor in hip OA pathology.

*DPT* is another gene remarkably expressed on OA chondrocytes in our study (FC = 58.28, *p* = 2.44E-06) that has not been reported previously as a differentially expressed gene. It is known to alter the function of TGFβ1 in endothelial cells and plays an important role in angiogenesis, fetal development, wound healing, and tumor metastasis [26]. Increased expression of *DPT* is observed in mesenchymal stem cells in chondrogenic differentiation [27]. Although the function of *DPT* in OA development and progression of OA is not clear, *DPT* expression is strongly inhibited by histone deacetylase (HDAC) 2 on human chondrocytes [18].

*IGFBP7* was also uniquely expressed in our OA samples compared to the previous studies. This gene encodes insulin-like growth factor-binding protein (IGFBP) 7, one of the IGFBP family proteins that regulate IGF action depending on tissue-specific regulation [28]. IGFBP inhibits IGF-1, which promotes cartilage proliferation and differentiation. Increased *IGFBP 3*, *4*, and *5* expression is observed in OA chondrocytes causing dysregulation of IGF-1 [29]. Previous studies have found the elevated expression of *IGFBP*-family genes in human OA cartilage than in healthy cartilage [30], however, no studies have reported of *IGFBP7* expression. Our study is the first report to demonstrate elevated *IGFBP7* expression in human OA chondrocytes.

During the onset and development of OA in the articular cartilage, various transcription factors (TF), e.g., *HIF2A* [31], *RUNX2* [32], and *SOX9* [33], regulate cartilage matrix-degenerating enzyme and inflammatory cytokine expression. These OA-related TFs play important roles in chondrocyte differentiation and OA progression. Meanwhile, these genes were not upregulated in OA chondrocytes in our study. Considering that expression patterns are known to vary by OA stage [34], *HIF2A*, *RUNX2*, and *SOX9* genes might not have been overexpressed in end-stage OA in our study.

In the present study, 26 TF genes were overexpressed in OA versus non-OA chondrocytes. Of these, *FOXO1* (forkhead-box protein O1) and *KLF2* (Krüppel-like factor 2) expression was prominently elevated in OA chondrocytes. *FOXO1* is widely expressed in various organs and regulates basic cellular physiological functions such as cell-cycle control, apoptosis, and glucose metabolism [35]. Akasaki et al reported *FOXO1* overexpression in the middle zone of OA cartilage as well as the difference in cellular localization of *FOXO1* between OA and healthy cartilage. Moreover, reduced *FOXO1* expression in articular cartilage increased susceptibility of chondrocyte death under oxidative stress [36]. Further investigation is necessary to clarify the function of *FOXO*1 overexpression in OA chondrocytes.

*KLF2*, a gene encoding Krüppel-like factor 2, was the most prominently expressed TF in OA chondrocytes in our study. *KLF2* is induced by fluid shear stress constituting the atherosclerosis pathway (http://genecodis.cnb.csic.es/). Central transcriptional regulator is also involved in matrix metalloproteinases (*MMPs*) regulation via NFκB in endothelial cells [37]. *MMPs* are well known catabolic genes that degrade ECM of articular cartilage [31]. Considering these facts, *KLF2* can be a potential regulator of expression of *MMP*s in chondrocytes, and related to OA onset and/or progression. *KLF2* expression was reportedly downregulated in human OA chondrocytes contrary to our results, wherein *KLF2* upregulation suppressed *MMP13* expression and ameliorated type II collagen degradation [38]. Although no information was provided on OA staging in Yuan’s report, this discrepancy could be a result of the difference in OA grade, as expression patterns of specific genes in chondrocytes depend on the stage of OA [39].

GO analysis found that ECM components were the most significantly enriched GO term (including 61 genes, *p* < 0.001, FDR = 0.069). Some microarray analyses also indicated increased expression of ECM-related genes in OA cartilage [16, 40]. Increased catabolism of the ECM in articular cartilage is playing a key role in the development and the progression of OA [41]. The high expression of ECM-related genes in OA chondrocytes is considered to be an “attempt” to remodel injured cartilage in response to altered cellular environment [42]. In this regard, our results support the findings in the previous studies.

Pathway analysis demonstrated the enriched ECM-receptor interaction, focal adhesion, and protein digestion and absorption pathways in the secondary OA chondrocytes. Cui et al [43] performed a meta-analysis based on 3 microarray databases for OA and healthy chondrocyte gene expression and confirmed that differentially expressed genes were enriched in ECM-receptor interaction and focal adhesion pathways. Based on enrichment map analysis for OA, they concluded that genes involved in these pathways can be essential for OA occurrence. Furthermore, these pathways affect non-traumatic necrosis of the femoral head [44] and are assumed to contribute to joint destruction. Protein digestion and absorption pathway is known to be associated with pancreatic neuroendocrine tumors and breast cancer as it is an essential protein degradation process for human nutrition homeostasis [45]. The relevance of this pathway to OA has not been clarified. In total 6 of 7 of upregulated pathways of Caucasian, primary OA were common with our Japanese, secondary data (indicated in bold in Table 4 and S3 Table), including the 3 pathways shown above. The interesting fact is that OA-related pathways are mostly common between secondary and primary hip OA, although the gene expression patterns in each pathway are unique to each other.

This study has several limitations. First, the gene expression patterns were obtained solely from the microarray analysis; therefore, the cause-and-effect relationship of each differentially expressed gene cannot be assumed. Second, the number of samples in the comparative study of primary OA was not large enough to generalize or determine the difference in expression patterns in each study. In addition, the difference of human races between primary and secondary OA in this study, can be a confounding factor. Further studies are needed to clarify the detailed network of the differentially expressed genes in hip OA chondrocytes as well as the difference between ethnic origins or etiologies of hip OA.

To summarize, to the best of our knowledge, this is the first study to investigate genome-wide changes in gene expression patterns in secondary hip OA chondrocytes on the secondary hip OA in Japanese population. *DPT*, *IGFBP7* and *KLF2* were newly found genes that were prominently overexpressed in secondary hip OA chondrocytes. ECM components were the most significantly enriched GO term according to GO analysis. Pathway analysis revealed the ECM-receptor interaction pathway as an OA-related pathway that is compatible with the previous report in primary OA. Although the OA-related pathways were similar between the secondary and primary OA, the contents of the differentially expressed genes were mostly heterogeneous.

## Supporting information

S1 Table Gene expression profiles. (XLSX)

S2 Table Functional enrichment analysis using a GSEA. (XLSX)

S3 Table The upregulated pathways in OA chondrocytes. (XLSX)
